# Process Development for the Continuous Manufacturing of Carbamazepine-Nicotinamide Co-Crystals Utilizing Hot-Melt Extrusion Technology

**DOI:** 10.3390/pharmaceutics17050568

**Published:** 2025-04-25

**Authors:** Lianghao Huang, Wen Ni, Yaru Jia, Minqing Zhu, Tiantian Yang, Mingchao Yu, Jiaxiang Zhang

**Affiliations:** 1 Key Laboratory of Marine Drugs, Ministry of Education, School of Medicine and Pharmacy, Ocean University of China, Qingdao 266003, China; 2 Pharmaceutical Products Research and Development Center, Marine Biomedical Research Institute of Qingdao, Qingdao 266137, China; 3 Material Characterization, Thermo Fisher Scientific, Shanghai 201203, China; minqing.zhu@thermofisher.com

**Keywords:** hot-melt extrusion, co-crystal, continuous manufacturing, process analytical technology tools, risk assessment, Box–Behnken design

## Abstract

**Objectives:** Hot-melt extrusion (HME) offers a solvent-free, scalable approach for manufacturing pharmaceutical co-crystals (CCs), aligning with the industry’s shift to continuous manufacturing (CM). However, challenges like undefined yield optimization, insufficient risk management, and limited process analytical technology (PAT) integration hinder its industrial application. This study aimed to develop a proof-of-concept HME platform for CCs, assess process risks, and evaluate PAT-enabled monitoring to facilitate robust production. **Methods:** Using carbamazepine (CBZ) and nicotinamide (NIC) as model compounds, an HME platform compatible with PAT tools was established. A systematic risk assessment identified five key risk domains: materials, machinery, measurement, methods, and other factors. A Box–Behnken design of experiments (DoE) evaluated the impact of screw speed, temperature, and mixing sections on CC quality. Near-infrared (NIR) spectroscopy monitored CBZ-NIC co-crystal formation in real time during HME process. **Results:** DoE revealed temperature and number of mixing sections significantly influenced particle size (D_50_: 2.0–4.0 μm), while screw speed affected efficiency. NIR spectroscopy detected a unique CC absorption peak at 5008.3 cm⁻¹, enabling real-time structural monitoring with high accuracy (R² = 0.9999). Risk assessment highlighted material attributes, process parameters, and equipment design as critical factors affecting CC formation. All experimental batches yielded ≥ 94% pure CCs with no residual starting materials, demonstrating process reproducibility and robustness. **Conclusions:** Overall, this work successfully established a continuous hot-melt extrusion (HME) process for manufacturing CBZ-NIC co-crystals, offering critical insights into material, equipment, and process parameters while implementing robust in-line NIR monitoring for real-time quality control. Additionally, this work provides interpretable insights and serves as a basis for future machine learning (ML)-driven studies.

## 1. Introduction

To improve efficiency and target specificity, new drug molecules are frequently designed with complex structures or enhanced with sophisticated functional groups [1]. However, such complex structures and added functional groups usually results in poor water solubility and permeability, leading to reduced oral bioavailability and complicated drug development [2,3]. So, improving solubility of poorly soluble drugs has long been an important concern in the area of drug development. Poor drug solubility remains a key pharmaceutical challenge, affecting ~40% of approved drugs and 90% of development-stage candidates, with many additionally limited by permeability issues and suboptimal safety profiles [4,5,6,7]. Consequently, finding cost-effective and efficient drug delivery methods to improve solubilization, enhance permeation, and control drug release to boost bioavailability remains a significant challenge in formulation research [4,5].

Several techniques have been used to improve drug solubility, such as forming amorphous solid dispersions (ASD), nanoparticles, liposomes, and forming co-crystals [6,7,8,9,10,11,12]. However, ASD, liposomes, and nanoparticle techniques have disadvantages such as physical instability, recrystallization, long-term stability, aggregation, and potential toxicity concerns [11,13,14,15]. So, forming CCs presents a unique and valuable solution to enhance the solubility, dissolution rate, and bioavailability of poorly water-soluble drugs [16]. Generally, CC is a type of crystalline material comprising two or more distinct molecules, one of which serves as the active pharmaceutical ingredient (API), assembled in a specific stoichiometric ratio within a shared crystal lattice, held together by non-ionic and non-covalent intermolecular interactions [17,18,19,20]. In addition to improving the bioavailability of low-solubility drugs, CC has many other advantages, including improved stability and oral bioavailability [21,22], tailoring physicochemical properties [23], and synergistically enhancing drug activity [24]. Notably, CCs can exhibit polymorphic behavior, which is the formation of distinct crystalline forms due to variations in molecular packing, hydrogen-bond networks, or stoichiometry within the same multicomponent system [25]. This CC polymorphism can significantly impact dissolution kinetics and physical stability; for example, a specific CC polymorph might combine high solubility with reduced hygroscopicity, optimizing formulation performance [25,26]. Furthermore, polymorphism represents a broader and complementary strategy for solid-state property optimization [26,27]. Polymorphs often exhibit marked differences in solubility, dissolution rate, and stability, allowing formulation scientists to tailor properties to specific needs; metastable polymorphs may enhance dissolution for rapid drug release, while thermodynamically stable forms can mitigate recrystallization risks during storage, addressing limitations common in amorphous or nano systems [27,28,29]. Various techniques are typically utilized for CC, such as solvent evaporation, mechanochemistry, and melt crystallization [30,31].

Hot-melt extrusion (HME) may be one of the most significant approaches in CC preparation that can achieve industrial-scale production [30,32]. HME has been widely used in a broad spectrum of applications in the pharmaceutical field [30,32,33]. Several drug products made by HME have been approved by the US Food and Drug Administration (FDA) and used clinically, suggesting the huge potential of HME in pharmaceutical manufacturing [34]. Continuous co-crystallization via HME is using heat to melt one or both initial materials then the mechanical forces will be applied for the molecular level mixing, thus forming the co-crystals during the cooling and recrystallization process, which made it become an efficient approach to improve the thermodynamic solubility of poorly water-soluble drugs [34]. The preparation of CCs via HME has been part of efforts towards the adaptation of continuous manufacturing (CM) principles in the pharmaceutical industry [35,36]. Compared to conventional approaches, HME is an organic solvent-free, cost-effective, continuous, high-quality, and green technology utilized for various formulations [32,37,38]. In this work, the initial materials and process parameters including temperature profiles, screw configurations, screw speed, and residence time may significantly affect the formation of co-crystals motors [39,40,41]. Therefore, it is essential to comprehensively understand and optimize the process by considering all potential attributes following the “Quality by Design (QbD)” principles.

Additionally, PAT tools are predominantly utilized for continuous processes, enabling real-time detection of product progress [42,43,44,45,46]. Such tools include near-infrared (NIR) spectroscopy, ultraviolet spectroscopy, and Raman spectroscopy, which the facilitate dynamic monitoring of material properties and process efficiency. Employing PAT tools for real-time monitoring of CC formation holds greater significance because the HME process encompasses solid-state transformation and has a substantial impact on the properties and quality of the final product [32,47]. Moradiya et al. devised a method for CC formation by installing NIR monitoring have been widely used for obtaining the CC formation during HME process [48,49]. Real-time monitoring with PAT allows for the timely adjustment and control of the process to ensure the desired CC qualities. Furthermore, recent advancements in machine learning (ML) have shown promise in pharmaceutical process development, such as predicting the impact of HME parameters on product quality or enabling real-time process optimization via PAT integration [50,51,52,53]. Consequently, considering the HME’s structural features and CC reaction conditions, carefully choosing the process analysis mode, deeply understanding drug CC interactions, and optimizing the process are essential to obtain high-quality and stable pharmaceutical CCs.

In this work, a continuous co-crystallization processes using HME has been successfully established. The influence of various risk factors was evaluated through a risk assessment studies employing HME-prepared CC of carbamazepine (CBZ) and nicotinamide (NIC). Based on the screening studies and Box–Behnken design, the critical materials attribution and process parameters in the HME process were analyzed and optimized. The design space and control strategy including manipulating molar ratio (CBZ: NIC), feeding rate, screw configuration, screw speed, and heating rate on the yield and particle size distribution were also obtained. In-line PAT were implemented, with an NIR probe used to monitor the formation of CBZ-NIC and quantification of the amounts of the CCs. This study provides valuable perspectives on the role of the key parameters of the CC of CBZ and NIC in the HME process and presents an effective path for high-throughput manufacturing of co-crystallization in the pharmaceutical industry.

## 2. Materials and Methods

### 2.1. Materials

Carbamazepine (CBZ, 99% pure) and nicotinamide (NIC, 99% pure) were chosen as model compounds for evaluating of the continuous co-crystallization process by HME. CBZ possesses five polymorphs (I, II, III, IV, and V), and form III is the most stable at room temperature (RT) [54,55]. CBZ and NIC were purchased from Macklin Biochemical Co., Ltd. (Shanghai, China). The form of CBZ was determined to be type III by X-ray powder diffraction (PXRD) and Fourier transform infrared spectrometer (FTIR). Methanol, ethanol, acetonitrile, and acetone were purchased from Macklin Biochemical Co., Ltd. (Shanghai, China). All other chemicals, solvents, and reagents used in this work were either analytical grade (purity ≥99.9%, ≤0.1% impurities) or high-performance liquid chromatography (HPLC) grade (purity ≥99.99%, ≤0.01% impurities).

### 2.2. Risk Assessment Study

#### 2.2.1. Ishikawa Diagram

**Figure 1:**
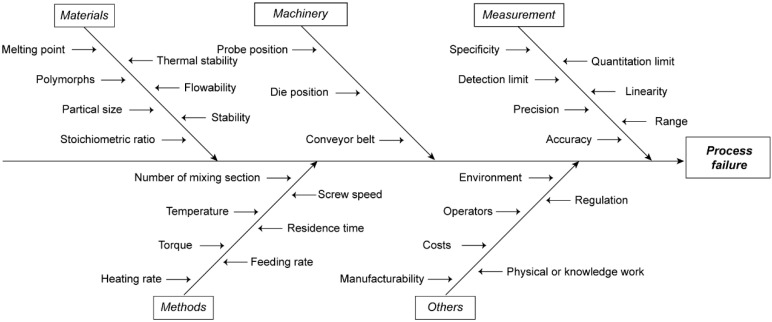
Ishikawa diagram showing potential risk factors that may lead to process failure in HME process.

An Ishikawa diagram (Figure 1) has been summarized to show possible factors for process failure during HME. The following five attributions of risks have been identified during process development: (1) Materials, (2) Methods, (3) Machinery, (4) Measurement, (5) Others. In this work, we will discuss the first four area in details as well as some case studies that we have performed.

#### 2.2.2. Process Development

##### Preparation of CBZ and NIC CC

In this work, the CC of CBZ and NIC were prepared by solvent evaporation methods (CC-SE), batch melting method (CC-MM), and continuous HME. Briefly, for CC-SE, a quantitative sample was taken according to the specified molar ratio (1:1, 1:2, and 2:1) of CBZ and NIC, and then added to 20 mL of an organic solvent. Four different organic solvents, including methanol, ethanol, acetonitrile, and acetone, were employed to prepare the CBZ-NIC CC. Subsequently, the solution was heated at 60 °C until completely dissolved within 2 h, and then placed in a fume hood at room temperature (RT) for cooling and volatilization.

The melting method (MM), a solvent-free approach, involves taking a sample with a specific molar ratio (1:1, 1:2, and 2:1) and placing it in the NLH-600 CG hot press machine (TianJin Nuolei Xinda Technology Co., Ltd., Tianjin, China). Set the target temperature and heat for 10 min without setting extra pressure, and then cool it at RT.

A Thermal Fisher Scientific 11-mm corotating twin screw extruder (Process 11 HYG) (Thermal Fisher Scientific, Shanghai, China) with eight individual heating zones was used for the preparation of CBZ and NIC CC. Throughout the extrusion process, melting temperature, torque, and die pressure were closely monitored and recorded. Detailed experiments methods were mentioned in Section “Design of Experiments ” and Section 2.2.3.

##### Screw Configuration

**Figure 2:**
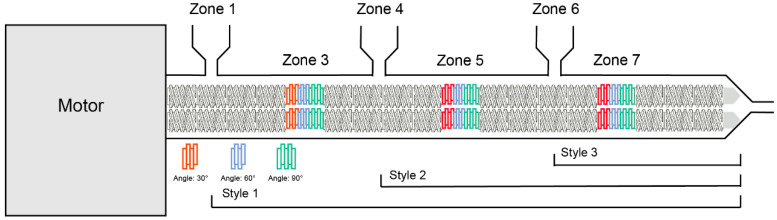
The demonstration of screw design and extrusion set-ups.

The screw configuration employed in this study is illustrated in Figure 2. The materials entered from zone 1 and then passed through varying numbers of engaging sections. The L/D ratio of the full screw was 40 and three individual kneading sections (length to diameter ratio is 6.5) were set at zone 3, 5, and 7.

##### Screening of Critical Impact Factors

It has been reported that several factors, including the heating rate, screw speed, number of mixing sections, and temperature, can potentially have an impact on the formation of CCs and extruded torque [56,57,58,59]. So, the following factors were investigated in this work.

Heating Rates: The effect of the heating rate on the formation of the CBZ-NIC CC was explored through the use of PLM. Specifically, the heating rates were set at 5, 20, and 50 °C/min, respectively, and the melting phenomena of each group were carefully observed.

Temperature: The influence of temperature on CC formation was investigated by means of the melting method (CC-MM). The temperature of MM was set at 110 °C, 130 °C, 150 °C, 165 °C, and 170 °C, respectively. The samples were heated for 5 min and then cooled to RT.

Screw Speed and Number of Mixing Sections: The impact of screw speed and number of mixing sections on the formation of the CBZ-NIC CC was explored by HME. HME was conducted with the style 3 screw configuration, where the materials were fed from zone 6. One batch was conducted with one kneading section and the other batch did not. The screw speed was set at 50 and 100 rpm.

##### Design of Experiments (DoE)

**Figure 3:**
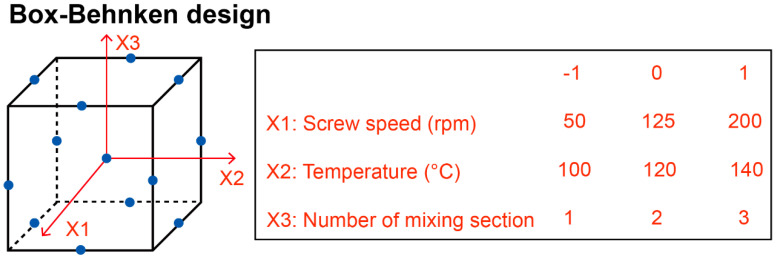
Box–Behnken design of HME parameters. Blue dots represent the experimental points in the design.

The Box–Behnken design was used to understand the correlation between impact factors and the HME process, which incorporated 12 design groups for the purpose of evaluating the impacts of various variables, and the center point was repeated for every 5 batches to check the reproducibility of the HME process. During the preliminary investigations, the heating rate, screw speed, screw design, and barrel temperature had already been optimized. Consequently, the screw speed (X1), temperature (X2), and the number of mixing sections (X3) were chosen as the independent variables to further optimize the formulation (Figure 3). The formation of CCs, particle size, and yield were used as the output for understanding the correlation and optimization of the HME process. Statistical computations were performed using Design Expert 8.0.6 software (Stat-Ease, Inc., Minneapolis, MN, USA).

#### 2.2.3. Continuous HME Process Set-Up

The system is composed of an extruder (left side) and a NIR spectrometer (right side). The extruder was equipped with an automatic feeder to ensure the addition of materials. Feeding rate, screw speed, and temperature were precisely controlled, and process conditions such as torque and real temperature were monitored as well. The process temperature was precisely adjusted through a cooling operation achieved by an external water-bath circulator.

**Figure 4:**
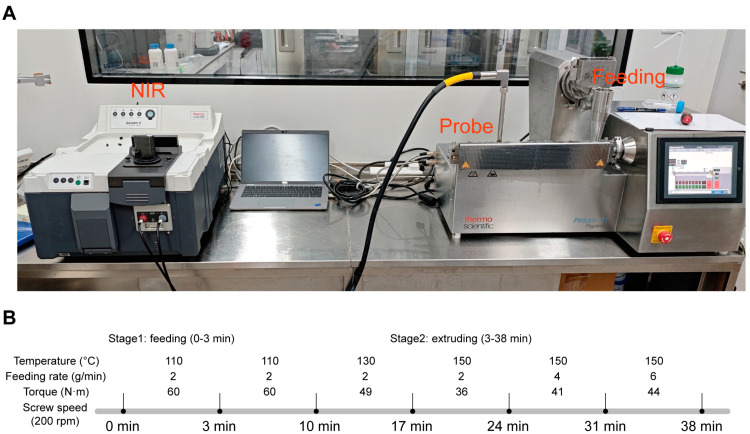
Schematic of the continuous system set-up (**A**) and PAT process (**B**).

An Antaris II NIR spectrometer (Thermo Scientific, Basingstoke, UK) was utilized for the in-line detection to realize the detection of the contents of CBZ and NIC CC. The instrument has the capability to integrate multiple modules, including the integrating sphere analyzer module, transmission analyzer Module, the optical-fiber-probe module, and the diffuse-transmission-detection module. Each of these detection modules utilizes its own high-sensitivity InGaAs detector for enhanced performance and accurate measurements. In this work, the integrating sphere analyzer module was employed and a high temperature probe was inserted into the zone 7 (Figure 4A). The probe with a sapphire window was installed in a threaded port at the middle of the extruder barrel. It was flush-mounted, enabling the sensor tip to make contact with the extruded material. The probe was configured to gather spectra every 20 s. Each sample reading averaged 32 individual spectra at a resolution of 8 cm^−1^. These spectra were scanned within the 4000–10,000 cm^−1^ wave-number range (2500–1000 nm wavelength) by using Omnic 9 (Thermo Scientific, Basingstoke, UK) software.

As depicted in Figure 4B, the PAT process was partitioned into six distinct test conditions. For this process, the style1 screw configuration was selected and the feeding was conducted from zone 1. Throughout the process, the formation of the CC of CBZ and NIC was detected in real-time by NIR, and further analysis was computed through off-line PXRD.

#### 2.2.4. PAT Tools Implementation

##### Specificity

The CBZ, NIC, physical mixture (PM), and CC prepared by solvent evaporation methods (CC-SE) were subjected to off-line NIR detection. Detailed NIR and CC-SE experimental methods have been mentioned in Section “Preparation of CBZ and NIC CC” and Section 2.2.3.

##### Linearity and Range

CC-SE was mixed with PM in the ratios of 0%, 5%, 25%, 50%, 75%, and 100% *w*/*w*. Subsequently, NIR was employed for detection purposes to determine the CC content of NIC and CBZ, and a linear regression equation was established. Detailed NIR experimental methods have been mentioned in Section 2.2.3.

##### Precision and Accuracy

Samples were analyzed with three replicate measurements by off-line NIR spectroscopy, and the associated errors were calculated. The CC content was then computed in accordance with the established formula. A total of 20% of CBZ-NIC CC was precisely weighed and mixed with 80% of PM, and then the offline NIR spectrum was collected to validate the accuracy of the NIR measurement.

##### The Yield of the CC

For each experiment, the yield was calculated as follows:

$$Yield=\frac{M_{feed}-M_{sample}}{M_{feed}}*100\%$$

where the *M_feed_* is the mass of the feeding sample (g), and *M_sample_* is the mass of the EXT (g).

#### 2.2.5. Off-Line Characterization

Yield, particle size, and distribution, as well as the polymorphic form, are major design considerations for conventional crystallization process. All samples obtained from the HME were evaluated using off-line characterization methods.

##### Thermogravimetric Analysis (TGA)

A TGA (Netzsch TG 209 F3, NETZSCH Geratebau GmbH, Selb, Bavaria, Germany) was utilized to acquire the thermal degradation information of CBZ, NIC, and PM. Samples weighing 5–10 mg were placed on the sample pan and then heated from 20 °C to 400 °C under the condition of being purged with ultra-purified nitrogen. Microsoft Excel (Version 2310 Build 16.0.16924.20054) was employed for data collection and analysis.

##### Differential Scanning Calorimetry (DSC)

A DSC 200 F3 equipment (NETZSCH Geratebau GmbH, Selb, Bavaria, Germany) was employed to obtain the melting properties of CBZ, NIC, PM, and CCs. A total of 5–15 mg of the samples were weighed and transferred to standard aluminum pans, which were then sealed with standard aluminum lids (DSC Consumables, Inc., Austin, Minnesota, USA). The analysis was carried out within a temperature range of 20 °C to 220 °C at a ramp rate of 20 °C/min.

##### Powder X-Ray Diffraction (PXRD)

The crystallinity of CBZ, NIC, PM, and CCs was explored by means of a benchtop PXRD instrument (D/max-2200PC, Rigaku Corporation, Tokyo, Japan). Briefly, the samples were loaded onto the sample cells and then placed in the sample holder. In this holder, the samples were scanned within a 2θ angle range from 5° to 50° at a scan speed of 2°/min, with a scan step of 0.02° and a scan resolution of 0.0025. The current and voltage of the system were kept at 15 mV and 45 V, respectively, using Cu Kα radiation as the X-ray source. The collected data were presented as an overlay graph of 2θ versus intensity.

##### Hot-Stage Polarized Light Microscopy (PLM)

A CX40P polarized photomicroscope (Ningbo ShunYu Analytical Instrument Co., Ltd., Yuyao, China) integrated with a hot-stage (Linkam Scientific Instruments Ltd., Tadworth, Surrey, UK) was adopted to explore the melting behaviors of CBZ, NIC, PM, and CCs. The drug and the milled samples were uniformly distributed on a glass slide, and the extra powder was removed. The analysis was carried out at a ramp rate of 20 °C/min. Subsequently, a coverslip was placed on the sample slide, which was then positioned on the microscope stage for observation at 10-fold magnification. Images were taken by a dedicated digital camera (EP-SUF880, Quest Scientific Instruments Inc., North Vancouver, BC, Canada) under light and dark background conditions with the help of a 530-nm compensator (U-TP530, Olympus Corporation, Shinjuku City, Tokyo, Japan).

##### Fourier Transform Infrared Spectroscopy (FTIR)

All samples were analyzed by employing a Varian 600 series FTIR spectrophotometer (Agilent, Waltham, MA, USA). The sample powders were blended with dry KBr (IR, spectroscopy grade, Macklin Biochemical Co., Ltd., Shanghai, China) at a ratio of 1:100. Subsequently, these mixtures were compressed into semitransparent pellets under a pressure of 5 tons for 1 min. The scanning range was set from 400 to 4000 cm^−1^. Each spectrum was obtained through 32 scans with a resolution of 4 cm^−1^. The samples were examined in transmission mode, and Omnic 9 (Thermo Scientific, UK) software was utilized for data analysis.

##### Detection of Particle Size

Sample particle size measurements were carried out by employing a BT-2001 laser particle size analyzer (Bettersize, Dandong, China). The samples were dispersed into the feeding system. For the analysis, the refractive index of the particles was set at 1.28 and the General-Purpose model with the Irregular Shape Mode were adopted. Before adding the sample, the background light scattering signals of the system were measured. Each sample underwent three repeated measurements, and the collected data were analyzed using analyze software (Bettersize, Dandong, China, Version 7.2).

##### Scanning Electron Microscope (SEM)

The sample was directly adhered to the conductive adhesive. Subsequently, the Quorum SC7620 sputtering coater (Quorum, Laughton, East Sussex, UK) was employed to conduct gold spraying for 45 s. The sputtering target was platinum, and the gold spraying current was 5 mA. Thereafter, the MIRA 4 scanning electron microscope (TESCAN, Brno, Czech Republic) was utilized to photograph the morphology of the sample. The acceleration voltage was set at 3 kV, and the detector was the SE2 secondary electron detector.

## 3. Result and Discussion

### 3.1. Critical Materials Attributes

#### 3.1.1. Thermal Stability of Initial Materials

**Figure 5:**
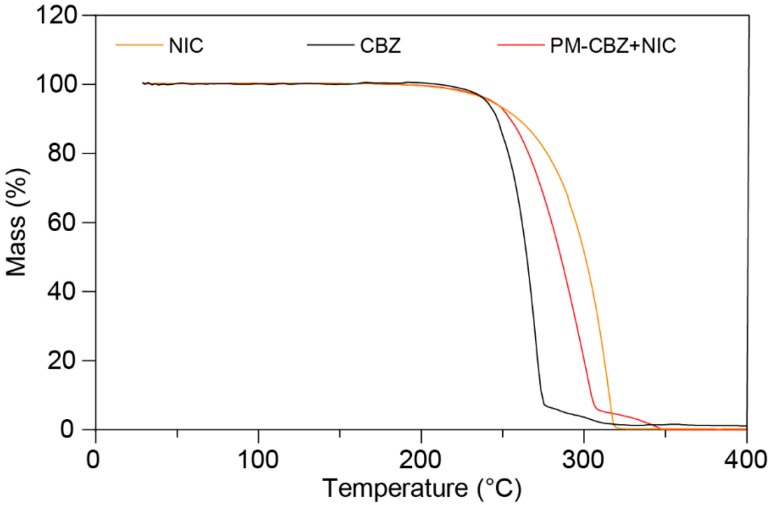
The thermogravimetric curves of CBZ, NIC, and PM.

During thermal processing, ensuring the thermal stability of materials is vital to avoid potential thermal degradation. A thermogravimetric analysis of CBZ, NIC, and PM was carried out. The samples were heated to 400 °C at a rate of 20 °C/min. As presented in Figure 5, raw CBZ, NIC, and PM demonstrated one-step thermal degradation starting at temperatures above 240 °C. Furthermore, because the temperature used in this work was below 175 °C, the process temperature was safe for the raw materials.

#### 3.1.2. Polymorphs of Initial Materials

**Figure 6:**
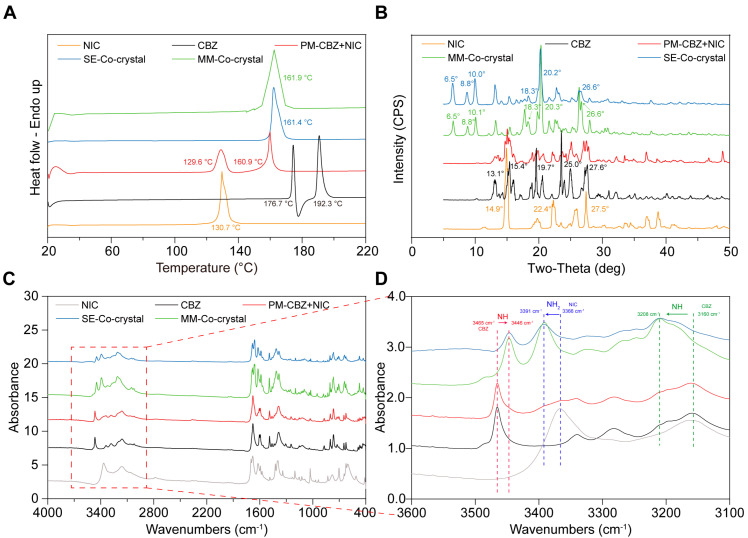
The characterization of CBZ, NIC, PM, and CC prepared by melting method (CC-MM) and solvent evaporation methods (CC-SE, prepared by ethanol). (**A**) DSC; (**B**) PXRD; (**C**,**D**) FTIR.

**Figure 7:**
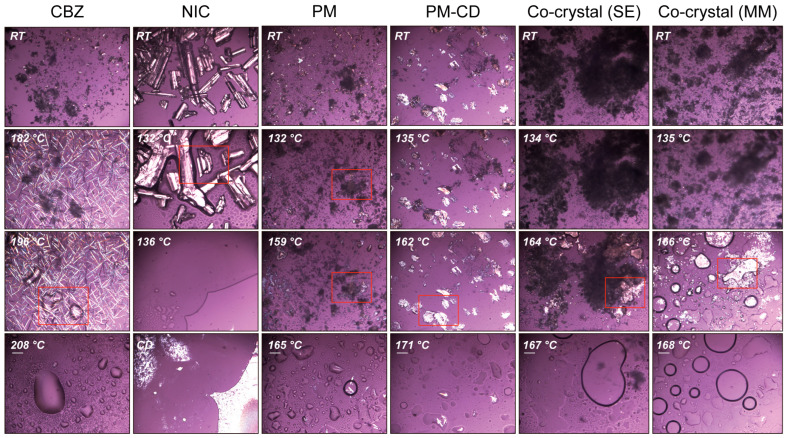
The PLM images of CBZ, NIC, PM, and CC prepared by the melting method (CC-MM) and solvent evaporation methods.

Form III is the most stable form of CBZ and has been widely used in pharmaceutical formulations [60,61]. Hence, form III CBZ crystals were used in this study. According to the relevant literature, form III CBZ exhibits a polymorphic transformation around 170 °C to form I and then ultimately melts at 193 °C [62,63,64,65]. DSC (Figure 6A, black curve) and PLM (Figure 6C, first column) matched the reported polymorph characterization of form III of CBZ. The melting point of NIC was determined to be 130.7 °C, which also matched the previously published literature. Additionally, the PM showed two distinct melting peaks at 129.6 and 160.9 °C in the DSC curve, which indicated the molten NIC rapidly formed CC with CBZ and then melted at around 160 °C. The same melting phenomenon was observed during the PLM studies (Figure 7, third column). The TG results of CBZ, NIC, and PM all exhibit single-step degradation profiles, which collectively suggest that the endothermic peaks observed in DSC and PLM correspond to crystalline phase transitions or melting processes rather than material decomposition or chemical structural alterations. In addition, the PLM sample of PM was cooled down to RT and reheated, and only one melting was observed started at around 160 °C, which indicated the formation of CCs by the melting method.

To confirm that NIC and CBZ formed CC by melting and solvent methods, DSC showed only one endothermic peak in both MM (Figure 6A, green curve) and SE (Figure 6A, blue curve) CCs. Additionally, there was no melting of individual CBZ or NIC observed in the SE and MM CCs, but an obvious endothermic peak near 162 °C was present (Figure 7). These thermal characteristics collectively confirm the successful formation of homogeneous CCs through both methodologies, eliminating the possibility of residual starting materials or PM.

The PXRD results in Figure 6B show that the characteristic peaks of NIC are 14.9°, 22.4°, and 27.5°, and those of CBZ are 15.4°, 19.7°, and 25°, as reported [61,66]. The characteristic peaks of both ingredients were observed in the PM group. New characteristic peaks, such as 6.5°, 8.8°, 10.0°, and 20.3°, emerged in the products prepared using the MM and SE methods, which matched the reported CBZ-NIC CCs. Additionally, as the FTIR results shown in Figure 6C, after NIC and CBZ are successfully prepared into a CC, the -NH peaks of CBZ shift from 3160 cm^−1^ and 3465 cm^−1^ to 3208 cm^−1^ and 3446 cm^−1^, respectively, and the -NH_2_ peak of NIC shifts from 3366 cm^−1^ to 3391 cm^−1^, which can be observed in Figure 6C,D. The same results were obtained during the infrared characterization of the products prepared by MM and the solvent evaporation method, and the peaks corresponding to NIC and CBZ did not appear at their original positions. Notably, the absence of original NIC and CBZ characteristic peaks in both MM and SE products confirms complete CC formation. These complementary solid-state analyses conclusively demonstrate the successful generation of phase-pure CBZ-NIC CCs through both synthetic routes, with no residual precursors or physical mixture components detected.

#### 3.1.3. Different Polymorphs of NIC- and CBZ CC and Stoichiometric Ratio

**Figure 8:**
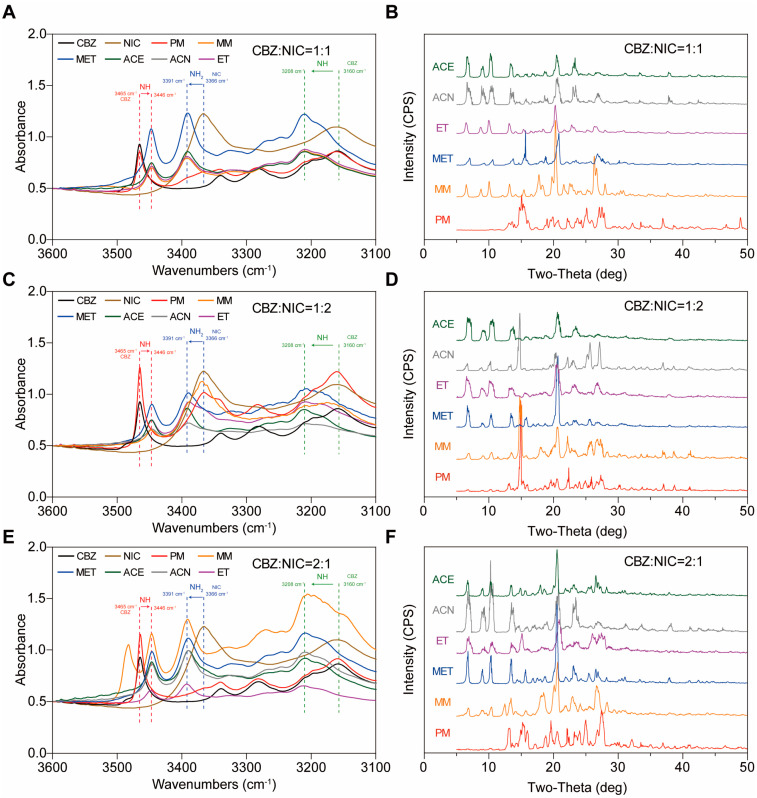
The FTIR and PXRD of CC with different molar ratios prepared by MM and solvent evaporation method in methanol (MET), ethanol (ET), acetonitrile (ACN), and acetone (ACE). The molar ratio of CBZ:NIC (**A**,**B**) was 1:1; (**C**,**D**) was 1:2; (**E**,**F**) was 2:1.

To determine whether there were different polymorphs of NIC-CBZ CC, MM, and SE with different solvents were used to prepare CC at different molar ratios. When the molar ratio of CBZ:NIC was 1:1, the FTIR and PXRD curves of the CC prepared with various solvents (MET, ACE, ACN, and ET) were consistent with CC-MM and CC-SE prepared by ET. As shown in Figure 8A,B, the corresponding groups had the same shifts, and new characteristic peaks also appeared at 6.5°, 8.8°, and 10.0° in the PXRD. The same form could be obtained using different solvents.

When the molar ratio of CBZ:NIC was 1:2, the CC prepared by MM had an obvious characteristic peak at 14.9° in the PXRD, which is the characteristic peak of NIC (Figure 8D). This indicates that both CC and a single NIC would simultaneously appear when using the MM. The same phenomenon was observed when acetonitrile was used as the solvent. This is because NIC may have a solvent effect with ACN, resulting in the precipitation of NIC due to reduce its solubility [67]. The other solvents yielded the same CC form of NIC and CBZ (Figure 8C,D).

When the molar ratio of CBZ:NIC was 2:1, the FTIR and PXRD patterns of CC prepared with various solvents were consistent with those prepared by the MM method and ET (Figure 8E,F). In the CC-MM, new peaks appeared at 3483.1 cm^−1^ in the FTIR and at 12.3° in the PXRD. This is because during the heating process, excess CBZ was simultaneously affected by the hydrogen-bonding force and heating, resulting in the transformation from form III to form I [68]. To sum up, there may be only one CC form of NIC and CBZ. However, when there is excessive CBZ or NIC, there may be remaining single components or polymorphic transformation. Therefore, in subsequent experiments, the molar ratio of CBZ to NIC was set to 1:1.

### 3.2. Critical Process Parameters

Several factors, such as the heating rate, screw speed, number of mixing sections, and temperature, have been reported to potentially influence the formation of CC and processibilities [68,69]. Hence, the aforementioned parameters are elaborated in detail in the following discussion.

#### 3.2.1. Single Factor Investigation of Batch Crystallization Process

##### Temperature

**Figure 9:**
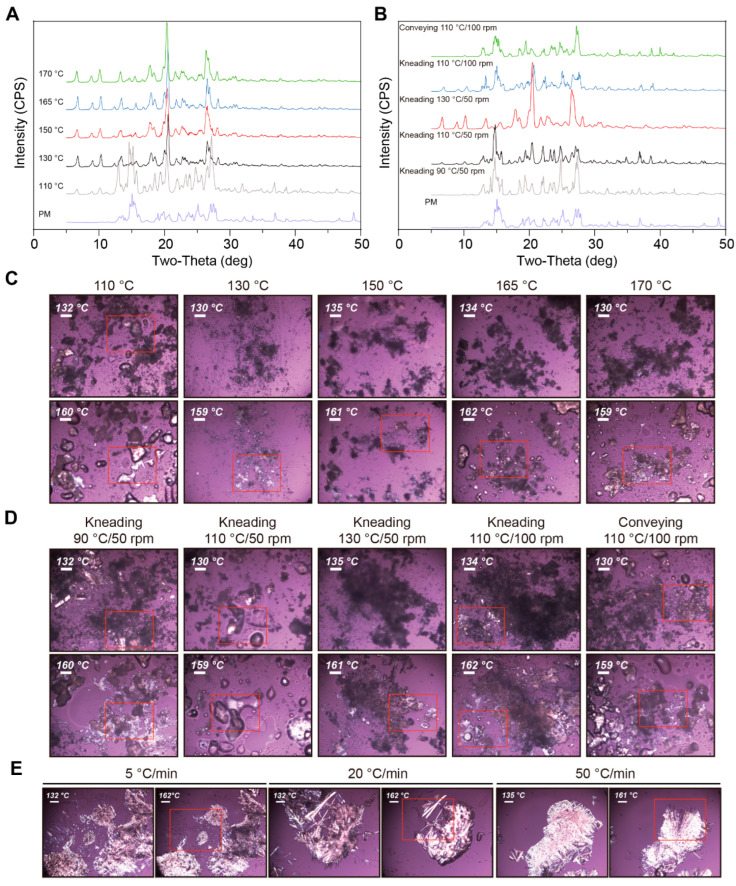
The factors potentially influencing the CC formation of NIC and CBZ during HME were screened. (**A**,**C**) The CC was prepared by MM with different temperatures; (**B**,**D**) prepared with different mixing section, screw speed, and temperature by HME; (**E**) prepared with different heating rates as 5, 20, and 50 °C/min by PLM. After being heated to 170 °C, it was cooled down to RT. Samples were detected by PXRD and PLM, respectively.

The influence of the process temperature was investigated using MM. CC of NIC and CBZ (molar ratio of 1:1) was prepared at 110 °C, 130 °C, 150 °C, 165 °C, and 170 °C during the MM process, respectively. As depicted in Figure 9A, except for the PM group, new characteristic peaks emerged at 6.5°, 8.8°, and 10.0° at the remaining process temperatures, thereby indicating the formation of CC at different temperatures. Nevertheless, at 110 °C the characteristic peaks of NIC and CBZ at 14.9° and 15.4° were clearly identified, indicating that the remining CBZ and NIC could not form CC. Figure 9C further reveals that the sample prepared at 110 °C exhibited a melting phenomenon at 130 °C, which is attributed to the melting of single NIC at this temperature. Moreover, melting NIC was not observed under the other process temperatures; thus, the authors concluded that the complete formation of CBZ-NIC CC was above 130 °C using the melting method.

Additionally, the heating rate is another aspect worthy of investigation. The PM samples were heated to 170 °C at the rates of 5, 20, and 50 °C/min on hot-staged PLM, respectively. Subsequently, they were cooled to RT for PLM analysis. The initial heating ramp exhibited the same melting phenomenon, as depicted in Figure 7. As depicted in Figure 9E, all the samples melted at approximately 160 °C, suggesting that all CBZ and NIC formed CC, which means that the heating rate has no significant influence on the preparation of CC. These comprehensive thermal analyses establish processing temperature as the critical determinant for complete CBZ-NIC crystallization, while revealing heating rate independence within the experimental parameter space.

##### Screw Configurations

HME was conducted to evaluate the impact of temperature, screw configuration, and screw speed on the CC preparation. The HME was conducted as described in section “Screw Speed and Number of Mixing Sections”. As shown in Figure 9B, no characteristic peaks of CC were observed at 90 °C with one mixing section at 50 rpm. Moreover, the screw speed cannot be increased because the unmolten CBZ and NIC contribute to the extremely high torque during the HME. At 110 °C, the characteristic peaks of CC, individual NIC, and CBZ were identified, while only the characteristic peaks of CC were detectable at 130 °C. This indicates that the extrusion process temperature is a crucial factor influencing CC formation. Notably, this temperature dependency supersedes mechanical energy inputs—screw speed variations (50→100 rpm) merely modulated melting kinetics rather than the final crystalline form. The 20 °C window between incomplete (110 °C) and optimal (130 °C) crystallization highlights the precision required in thermal process design for pharmaceutical CC manufacturing via HME.

When the screw speed was increased from 50 to 100 rpm, less unmolten NIC was observed, and melting at around 130 °C was observed at a higher screw speed at 110 °C, suggesting that the screw speed might affect the formation of the CC (Figure 9D). This suggests that higher screw speeds enhance the melting process of NIC through intensified shear heating and localized temperature rise, thereby indirectly influencing the formation of CC. When the mixing section was changed to a conveying section, no characteristic peaks of CC could be detected in the group, indicating that the mixing section has an impact on CC formation. This demonstrates that the shear-induced mixing in the mixing section is critical for CC formation—high shear forces and forced mixing likely trigger CC generation via molecular chain orientation or interfacial interactions, whereas the conveying section lacks sufficient shear energy to drive such structural evolution. This multivariate analysis establishes extrusion temperature as the dominant crystallization driver, while identifying screw design and rotational speed as critical secondary parameters modulating molecular diffusion and mechanical activation during thermoplastic crystallization.

##### Screw Speed and Residence Time

Samples prepared via MM at 110 °C exhibited detectable CC characteristic peaks (Figure 9A), likely due to prolonged thermal exposure enabling the formulation of CC. In contrast, HME (hot-melt extrusion) under similar temperature (110 °C) and screw speed (100 rpm) conditions resulted in negligible CC formation, as the extremely short residence time (~30 s) limited molecular reorganization. This highlights the critical role of residence time in CC formation: prolonged thermal exposure (as in MM) provides the kinetic energy required for structural ordering, while HME’s short processing window restricts this process. However, since residence time in HME is inversely correlated with screw speed (higher speed reduces material retention), the observed CC absence in HME may stem from both insufficient thermal duration and shear-induced dynamic effects. This implies that CC formation depends on a balance between thermal history (time-temperature integration) and shear-driven molecular activation, with screw speed acting as a dual-control parameter—modulating both residence time and shear intensity.

#### 3.2.2. Design of Experiment (DoE) Studies

##### The Formation of CC

**Figure 10:**
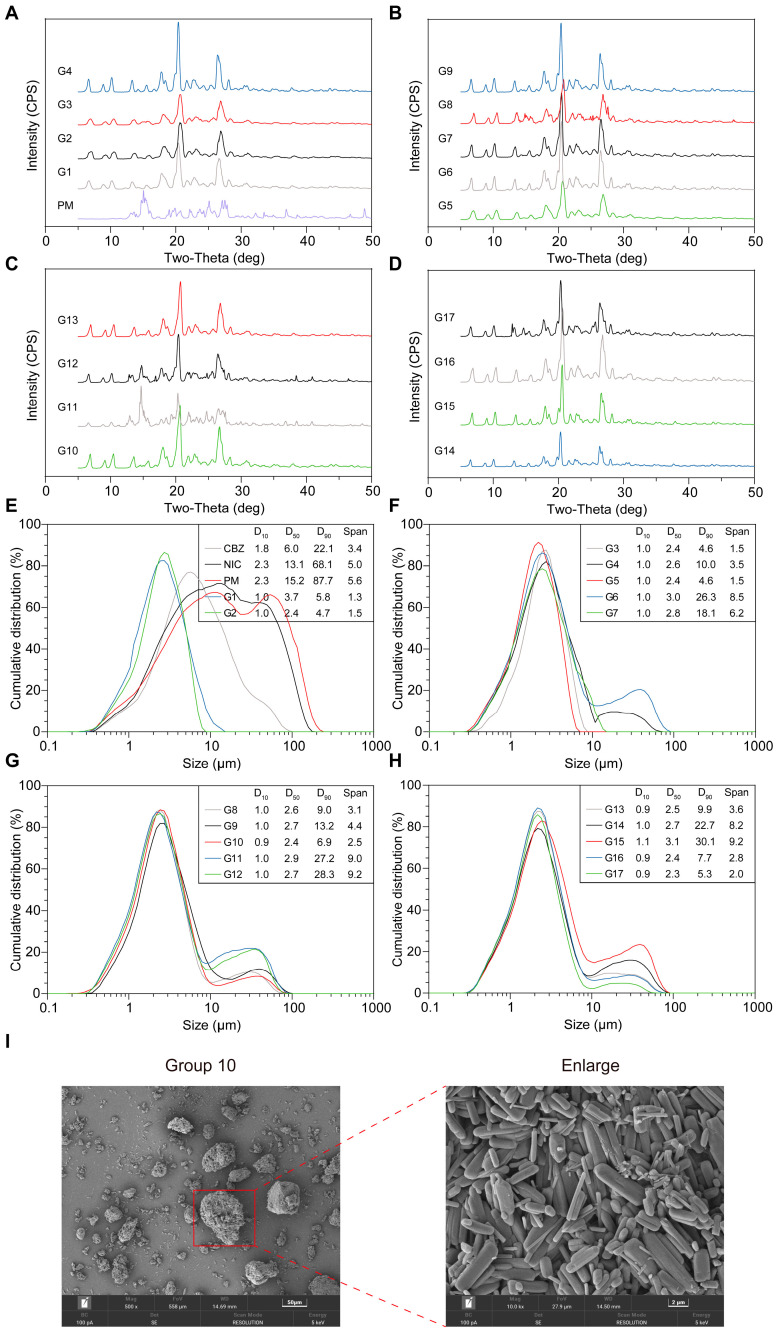
According to the design conditions specified by the DoE, the samples obtained through HME were analyzed using (**A**–**D**) PXRD and (**E**–**H**) particle size distribution. (**I**) SEM image of Group 10. Scale bar: 50 μm (left) and 2 μm (right).

To further analyze the significance of screw speed, temperature, and the number of mixing sections on the formation of CBZ-NIC CC, DoE was conducted based on the Box–Behnken design, with the purity and particle size of the CC being analyzed. As presented in Table 1, 17 trials were designed, among which there were five replicates of the central point (G4, 9, 10,13, and 16). The samples under the design conditions of 17 DoE groups were detected through PXRD and particle size distribution analysis. As shown in Figure 10A–D, all groups generated NIC and CBZ CC. Characteristic peaks were presented at 6.5°, 8.8°, 10.0°, etc., in all groups, and nearly all groups could only detect the CC component. This suggests that the selected parameter ranges (screw speed, temperature, mixing sections) lie within a stable design space where CBZ and NIC reliably form CC, regardless of minor variations in these factors. However, only groups 8, 11, and 12 were able to detect NIC and CBZ. Based on the PLM results (Figure S1), these three groups exhibited a melting phenomenon near 130 °C, which implies localized overheating or insufficient mixing under specific conditions, potentially disrupting complete CC conversion. These outliers highlight that while the overall process is robust, extreme parameter combinations (e.g., high temperature/low shear or vice versa) may transiently exceed the thermodynamic stability window of CC, leading to partial melting or phase separation.

Notably, the lack of statistical significance (*p* > 0.05 in response surface analysis, Table S1) for purity variations underscores that the tested parameter ranges were too narrow to expose critical thresholds for CC purity degradation. This indicates that the current design space is optimized for CC formation, but broader parameter exploration (e.g., higher screw speeds, lower temperatures, or extended mixing sections) is necessary to identify failure boundaries and refine process robustness.

##### Particle Size and Distribution

The particle size distributions of CBZ and NIC exhibit a relatively broader distribution. The distribution range of NIC presented no evident central tendency (SPAN = 5.0). Nearly all groups’ D_10_ values were about 1.0 μm, indicating various factors may not have a significant impact on D_10_ (Table S1). As depicted in Figure 10E,F, the results indicated that the D_50_ range was primarily 2.0–4.0 μm. As shown in Table 2, all the model of D_50_ *p*-values were <0.05, and original model has a higher R^2^ (0.8674). The strong model fit implies that D_50_ is predominantly controlled by temperature and mixing section interactions, despite screw speed showing non-significant individual effects. The analysis results demonstrated that screw speed had a non-significant effect on D_50_ (Table 2).

The results reveal that the main factor of D_90_ is the combined overall impact of temperature and the number of mixing sections with temperature having a more significant influence (*p*-value: 0.0002) (Table 3), likely because elevated temperatures promote particle agglomeration or partial melting, as supported by SEM observations in Group 10 (Figure 10I). SPAN is only influenced by temperature as a single factor (Table 4), highlighting that temperature-driven thermal expansion or softening governs particle size uniformity. Interestingly, only G1, G2, G3, G5, and G7 displayed a single peak, whereas all other groups had an additional peak after the main peak. Based on the SEM results, this might be due to the agglomeration of crystals rather than the actual crystal sizes (Figure 10I, group 10). This dichotomy suggests that specific temperature-mixing combinations (e.g., low temperature with limited mixing sections) exacerbate agglomeration, disrupting ideal size distributions. Consequently, the difference between D_90_ and SPAN was highly significant, and according to the response surface analysis this phenomenon was mainly associated with the temperature. In summary, the influence of the temperature and number of mixing sections on the particle size distribution is more significant than that of the screw speed. To clarify critical thresholds for agglomeration versus dispersion and explore the design space, expanding temperature ranges, testing extreme mixing section configurations, and incorporating torque-feedback controls will be conducted in future. Additionally, the effects of torque and feeding rate are discussed in Section 3.3 PAT Implementation.

#### 3.2.3. Reproducibility

Center points (screw speed = 125 rpm, T = 120 °C, 2 mixing zones) have been repeated in 5 batches (G4, G9, G10, G13, and G16), and the PXRD, DSC, and PLM analysis showed that all of these 5 groups were forming CBZ-NIC cocrystals. Additionally, the particle size analysis showed that the D_10_ and D_50_ were almost the same while D_90_ showed larger variations (Table 5), which may be due to the agglomeration mentioned in Section Particle Size and Distribution. Because of the agglomeration, the D_90_ and SPAN may be not applicable to represent the real process conditions compared to the D_10_ and D_50_. As a result, the reproducibility is acceptable because all these 5 groups formed cocrystals completely and have same D_10_ and D_50_.

### 3.3. PAT Implementation

#### 3.3.1. Instrument Set-Ups

The FDA initiated a regulatory framework for PAT implementation to improve the production process of the pharmaceutical industry in 2004. Motivated by the FDA, an increasing number of studies on the utilization of PAT in the HME process have been reported in recent years [42,43,44,45,46]. Researchers have applied Fourier transform infrared spectroscopy (FTIR) and UV/vis for supersaturation, however, ATR-FTIR has limited capability to distinguish the impurities and UV/vis was not adequate for high concentration [70,71]. NIR is an effective means for the in-line measurement of the content and stability of samples. However, the extrudates accumulated in the die section influence both the extrusion process and the in-line NIR analysis. The accumulation of extrudates generates extremely high pressure in the die, which stops the extrusion process and causes the loss of mobility of extrudates, resulting in the failure of real-time NIR detection. Additionally, if the NIR probe was installed above the conveying belt using the reflectance model, the signal was almost undetectable. To address the abovementioned problems, the DIE was initially removed and the NIR probe was placed in zone 7 (Figure 4A), facilitating stable real-time detection of the NIR spectrum of the sample.

#### 3.3.2. Specificity

**Figure 11:**
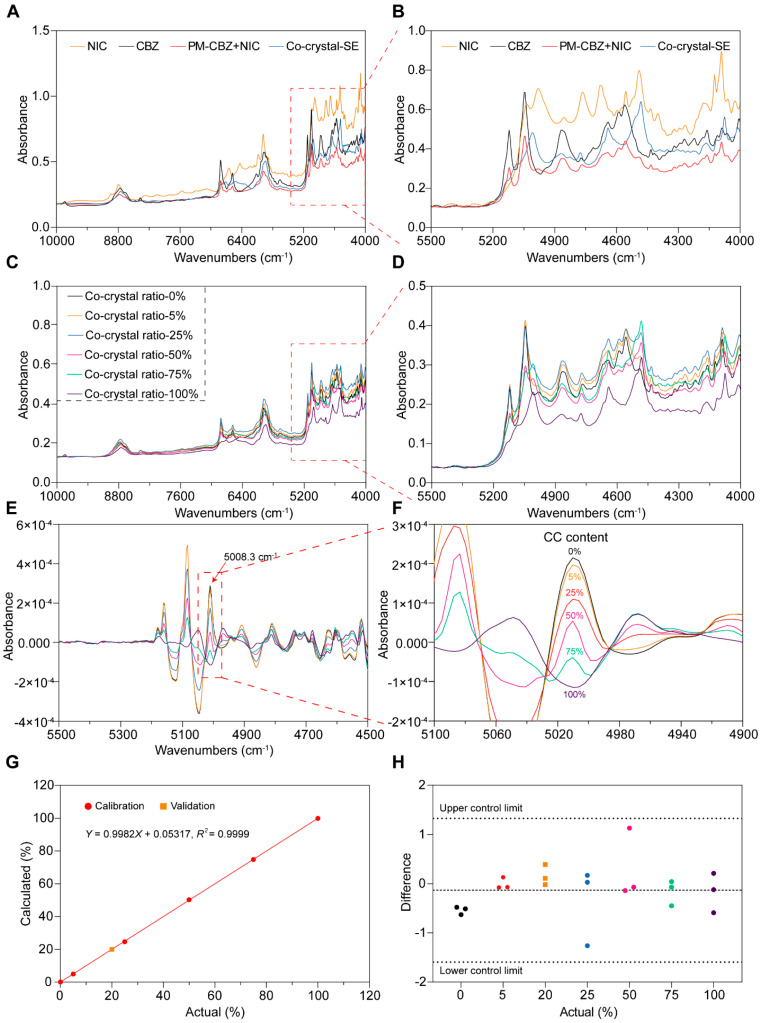
The samples were detected by NIR and the mathematical model was established. (**A**,**B**) NIC, CBZ, PM, and CC-SE. (**C**,**D**) PM and CC-SE were mixed in different ratios for detection; (**E**,**F**) second derivative of NIR spectra between 4500 and 5500 cm^−1^ wave-numbers; (**G**,**H**) the mathematical model and difference.

The offline NIR spectra of the powdered feedstock materials were measured to identify the peaks of CBZ, NIC, PM, and CC-SE, as shown in Figure 11A,B. The 5008.3 cm^−1^ peak of CC-SE was strong and unique, having great potential for CC measurement. When PM and CC were mixed in different proportions, characteristic peaks were detected using NIR spectroscopy (Figure 11C,D). However, NIR spectra are usually broad and overlapping, therefore, derivative mathematical treatment is required. A secondary derivative treatment was applied to these spectra (Figure 11E,F), and more obvious deviations from the PM spectra were found around the 4900–5300 cm^−1^ wave-number regions. So, the absorbances at 5008.3 cm^−1^ in the secondary derivative was used as the characteristic peak for the CBZ-NIC CC and for the further quantification studies as well.

#### 3.3.3. Linearity and Range

CBZ-NIC CC and PM in varying proportions mentioned in Section “Linearity and Range” were prepared, and their NIR spectra were obtained. The absorbances at 5008.3 cm^−1^ in the secondary derivative of collected spectra showed proportional to the weight ratio of CBZ-NIC CC (Figure 11G). Based on these data, a mathematical model was established (R^2^ = 0.9999) for the quantitative determination of the CC content in the subsequent HME process. This process is crucial because it helps in precisely analyzing the materials and enables better control in relevant applications.

#### 3.3.4. Precision and Accuracy

During the offline measurement, each sample were measured triplicated and the absorbance of peak 5008.3 cm^−1^ were listed in Figure 11H, where the upper control limit (UCL) and lower control limit (LCL) were calculated based on six sigma methodologies. As demonstrated in Figure 11H, the standard deviation measured samples were all between the LCL and UCL, which indicates that the NIR is precise enough for CBZ-NIC CC measurement. To validate the accuracy of the calibration curve, a known ratio of 20% *w*/*w* CBZ-NIC CC to PM mixture was precisely prepared and subjected to NIR analysis. As demonstrated in Figure 11G, the actual values perfectly fit the predicted models, which indicates that the prediction model is accurate for the quantification of CBZ-NIC CC.

#### 3.3.5. Monitoring CBZ-NIC CC Formation Using PAT

**Figure 12:**
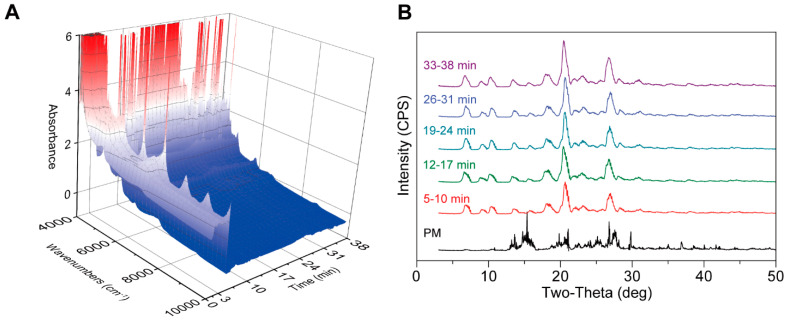
NIR can detect the generation of CC in-line during the PAT process. (**A**) NIR for in-line analysis (0-38 min). (**B**) PXRD for off-line analysis.

The inline NIR is presented in Figure 12A. During the 0–5-min period, which is the feeding stage, no CC signal was displayed, likely due to incomplete material homogenization or delayed crystallization kinetics prior to full melting and shear activation. In subsequent steps, essentially the same CC signals were obtained, indicating process stability once thermal and mechanical equilibrium is achieved. To further ascertain whether the sample obtained via PAT was CC, PXRD analysis was carried out. As shown in Figure 12B, all samples exhibited new characteristic peaks at 6.5°, 8.8°, and 10.0° for the CBZ-NIC CC, confirming that PAT-driven conditions reliably induce nucleation and growth of CC without phase impurities. As the feeding speed increased, the weights of the obtained samples showed a gradual upward trend. However, the final yield remained relatively stable at approximately 94%, which was due to the materials remaining in the barrel and errors during the feeding operation, suggesting that yield limitations are governed by equipment retention rather than intrinsic process inefficiency. Additionally, the torque increased with an increase in feeding speed (Table 6), consistent with higher material throughput elevating shear resistance in the extruder. It should be noted that torque is predominantly influenced by temperature. Specifically, as the temperature increased, the torque decreased by approximately 40% (Table 6), a result of reduced melt viscosity and softened material lowering shear stress. This study demonstrates the robust continuous manufacturing of CBZ-NIC CCs via PAT monitoring, with PXRD confirming crystalline purity despite variable feeding speeds. The temperature-dependent torque behavior and consistent 94% yield highlight the operational stability and scalability of the process, where thermal control mitigates mechanical load fluctuations, ensuring reproducible output. The remaining material must be improved by barrel design optimization to minimize yield fluctuations in future implementations, such as incorporating tapered screws or dynamic purge zones to reduce residual holdup.

While the study demonstrates robust PAT-enabled continuous manufacturing of CBZ-NIC CCs with stable yield and purity, potential limitations should be noted. SEM images (Figure 10I) revealed particle agglomeration during extrusion process, which may alter light scattering and affect the precision of NIR-based real-time quantification, particularly at high feeding speeds or with small shear force. Melt inhomogeneity due to insufficient shear could theoretically lead to localized variations in CC formation or residual starting materials, though this was not observed within the tested parameter space. These limitations highlight opportunities for future work, such as integrating in-line particle size monitoring to detect aggregation or expanding experimental ranges to assess extreme mixing conditions, which will further enhance the reliability and scalability of the HME process for continuous co-crystal production.

## 4. Conclusions

Through the current investigation, a continuous process of manufacturing CBZ-NIC CCs by HME was successfully established. As a promising approach, HME is highly scalable and avoids the use of excess organic solvents, rendering it cost-effective and environmentally friendly. Unlike traditional methods, this method employed current study shows the potential for consistent and efficient production of the CBZ-NIC CC. This study also comprehensively explored the factors influencing the formation of the CBZ-NIC CC.

According to the risk assessment studies, the investigation of material attribution indicates that the polymorph of the initial materials might not affect the CC formation of CCs because the ingredients melt and recrystallize during the HME process. The molar ratio of the initial materials may not affect the formation of the CBZ-NIC CC because only one CC polymorph was processed. However, the molar ratio affects the purity because the remaining excess CBZ or NIC is considered an impurity in the final products.

Process parameters, such as temperature, screw speed, and number of mixing sections, were investigated. According to the correlation analysis, the temperature and number of mixing sections had a significant influence on the particle size of the CBZ-NIC CC prepared by HME, and a formula for predicting the particle size was successfully established. In addition, the purity of the CC in the DoE results was almost 100%, probably because the selected ranges were within the design space. By precisely controlling these parameters, a better control of the CC formation process can be achieved. This innovation shows the potential for adjusting the properties of CC, such as purity and particle size distribution, by modifying process parameters, paving the way for the development of high-quality pharmaceutical products.

Real-time detection of CBZ-NIC CC content during the HME process was successfully performed using NIR. The characteristic peak at 5008.3 cm^−1^ was selected according to the off-line NIR analysis, and a mathematical model for determining the CC content was successfully developed. The accuracy, precision, specificity, linearity, and applicable range of these models were comprehensively investigated. The use of PAT with NIR spectroscopy for in-line monitoring of the CC formation process was successfully demonstrated. This allows real-time detection and adjustment during CC manufacturing. These findings enhance understanding and offer flexibility to create optimized drug delivery systems or materials with improved properties, meeting the specific needs of the pharmaceutical industry. Overall, this work successfully established a continuous process for co-crystallization using HME and the manufacture of CBZ-NIC CCs. This work also provided valuable insights into the critical material, instrumentation, and process attributes of the process, and also established robust monitoring using in-line NIR techniques. Additionally, the current study focuses on establishing a foundational HME platform and risk assessment framework using traditional design of experiments (DoE), which provides interpretable insights and serves as a basis for future ML-driven studies. Subsequent work will explore the integration of ML with PAT tools to enhance process robustness and dynamic control.

## Figures and Tables

**Table 1:** Box–Behnken design of 17 groups of HME parameters.

Group	Screw Speed, S (rpm)	Temperature, T (℃)	Number of Mixing Sections, N	D_10_ (μm)	D_50_ (μm)	D_90_ (μm)	SPAN	No Detectable Residual CBZ or NIC Peaks
1	125.00	140.00	3.00	1.0	3.7	5.8	1.3	Yes
2	200.00	140.00	2.00	1.0	2.4	4.7	1.5	Yes
3	125.00	140.00	1.00	1.0	2.4	4.6	1.5	Yes
4	125.00	120.00	2.00	1.0	2.6	10.0	3.5	Yes
5	50.00	140.00	2.00	1.0	2.4	4.6	1.5	Yes
6	50.00	120.00	3.00	1.0	3	26.3	8.5	Yes
7	200.00	100.00	2.00	1.0	2.8	18.1	6.2	Yes
8	50.00	120.00	1.00	1.0	2.6	9.0	3.1	No
9	125.00	120.00	2.00	1.0	2.7	13.2	4.4	Yes
10	125.00	120.00	2.00	0.9	2.4	6.9	2.5	Yes
11	125.00	100.00	1.00	1.0	2.9	27.2	9	No
12	50.00	100.00	2.00	1.0	2.7	28.3	9.2	No
13	125.00	120.00	2.00	0.9	2.5	9.9	3.6	Yes
14	125.00	100.00	3.00	1.0	2.7	22.7	8.2	Yes
15	200.00	120.00	3.00	1.1	3.1	30.1	9.2	Yes
16	125.00	120.00	2.00	0.9	2.4	7.7	2.8	Yes
17	200.00	120.00	1.00	0.9	2.3	5.3	2	Yes

**Table 2:** The mathematical models of correlation between the individual factors and D_50_.

	S	T	N	S·T	S·N	T·N	S^2^	T^2^	N^2^
*p*-value	0.8598	0.7248	0.0040	0.8029	0.3343	0.0060	0.5436	0.2608	0.0177
Significance	NS	NS	***	NS	NS	**	NS	NS	*
Code Equation	D_50_ = 7.7917 − 3.1347·N+ 0.0188·T·N+ 0.2931·N^2^								

Significance: NS *p* > 0.05, * *p* < 0.05, ** *p* < 0.01, *** *p* < 0.005. Units: S (rpm), T (°C), N (unitless), D_50_ and D_90_ (μm), SPAN (unitless).

**Table 3:** The mathematical models of correlation between the individual factors and D_90_.

	S	T	N	S·T	S·N	T·N	S^2^	T^2^	N^2^
*p*-value	0.3892	0.0003	0.0275	0.7282	0.2640	0.4662	0.8835	0.2177	0.3930
Significance	NS	***	*	NS	NS	NS	NS	NS	NS
Code equation	(D_90_)^−1^ = −0.3073 + 0.0041·T − 0.0333·N								

Significance: NS *p* > 0.05, * *p* < 0.05, *** *p* < 0.005. Units: S (rpm), T (°C), N (unitless), D_50_ and D_90_ (μm), SPAN (unitless).

**Table 4:** The mathematical models of correlation between the individual factors and SPAN.

	S	T	N	S·T	S·N	T·N	S^2^	T^2^	N^2^
*p*-value	0.5032	0.0002	0.1671	0.8191	0.4279	0.6912	0.5733	0.0565	0.7827
Significance	NS	***	NS	NS	NS	NS	NS	NS	NS
Code equation	(SPAN)^−1^= −1.3544 + 0.01416·T								

Significance: NS *p* > 0.05, *** *p* < 0.005. Units: S (rpm), T (°C), N (unitless), D_50_ and D_90_ (μm), SPAN (unitless).

**Table 5:** Center points groups of HME parameters.

Group	Screw Speed, S (rpm)	Temperature, T (℃)	Number of Mixing Sections, N	D_10_ (μm)	D_50_ (μm)	D_90_ (μm)	SPAN	Purity (100%)
4				1.0	2.6	10.0	3.5	Yes
9				1.0	2.7	13.2	4.4	Yes
10	125.00	120.00	2.00	0.9	2.4	6.9	2.5	Yes
13				0.9	2.5	9.9	3.6	Yes
16				0.9	2.4	7.7	2.8	Yes
		Average		0.94	2.52	9.54	3.36	/
	S.D.			0.05	0.13	2.45	0.74	/

**Table 6:** The results of the various stages of the PAT process.

Time (min)	Temperature (℃)	Feeding Rate (g/min)	Forming CC	Weight (g)	Yield (%)	Torque (N·m)
5–10	110	2	Yes	9.37	93.7	60
12–17	130	2	Yes	9.42	94.2	49
19–24	150	2	Yes	9.41	94.1	36
26–31	150	4	Yes	18.78	93.9	41
33–38	150	6	Yes	28.46	94.9	44

## Data Availability

The raw data supporting the conclusions of this article will be made available by the authors on request.

## Supplementary Materials

The following supporting information can be downloaded at: https://www.mdpi.com/article/10.3390/pharmaceutics17050568/s1, Table S1: The fitting models of the correlation between individual factors (screw speed, temperature, and number of mixing sections) and response (Purity, D50, D90 and SPAN); Figure S1: According to the design conditions specified by the DoE, the products were analyzed by PLM; Figure S2: PLM images of pure materials. (A&B) show CBZ during a heating-cooling-reheating cycle; (C&D) show NIC during a heating-cooling-reheating cycle.

## Abbreviation

CC: Co-crystal; HME, hot-melt extrusion; FDA, Food and Drug Administration; PAT, process analytical technologies; CM, continuous manufacturing; NIR, near-infrared; FTIR, Fourier transform infrared spectrometer; MM, melting method; SE, solvent evaporation method; RT, room temperature; DoE, design of experiment; TGA, themalgravmatric analysis; DSC, differential scanning calorimetry; PXRD, X-ray powder diffraction; PLM, polarized light microscopy; SEM, scanning electron microscope; MET, methanol; ET, ethanol; CAN, acetonitrile; ACE, aceton; UCL, upper control limit; LCL, lower control limit.
