# Supplementary material for: Process Development for the Continuous Manufacturing of Carbamazepine-Nicotinamide Co-Crystals Utilizing Hot-Melt Extrusion Technology
Source: Pharmaceutics. 2025 Apr 25;17(5):568. doi: 10.3390/pharmaceutics17050568 (PMC12115052; doi:10.3390/pharmaceutics17050568)
Supplement: Supplementary file 1 [file pharmaceutics-17-00568-s001.zip › pharmaceutics-3558410-supplementary.pdf]

Supplementary material

Table S1. The fitting models of the correlation between individual factors (screw speed, temperature, and number of mixing sections) and response (Purity, D_{50}, D_{90} and SPAN).

response	Model	R^2	*p-value*	response	Model	R^2	*p-value*
Purity	Original	0.7076	0.3317	D_{90}	Original	0.7942	0.0807
	Square Root	0.6956	0.2302		Square Root	0.8207	0.0540
	Natural log	0.6839	0.2526		Natural log	0.8468	0.0338
	Inverse Sqrt	0.6724	0.2752		Inverse Sqrt	0.8715	0.0196
	Inverse	0.6614	0.2974		Inverse	0.8940	0.0107
D_{50}	Original	0.8674	0.0217	SPAN	Original	0.8234	0.0517
	Square Root	0.8641	0.0234		Square Root	0.8392	0.0391
	Natural log	0.8599	0.0257		Natural log	0.8573	0.0498
	Inverse Sqrt	0.8550	0.0286		Inverse Sqrt	0.8775	0.0169
	Inverse	0.8495	0.0320		Inverse	0.8980	0.0095

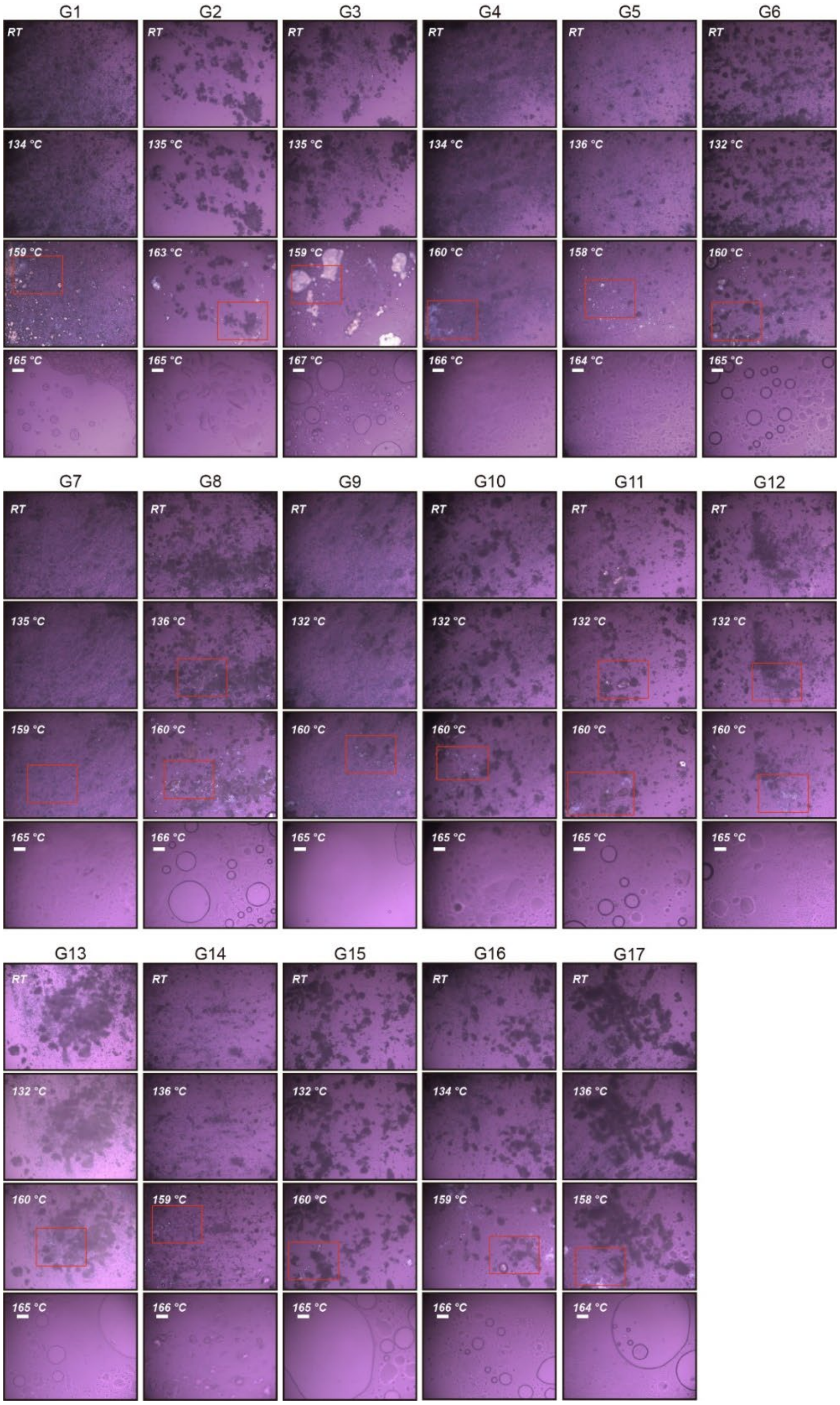

Figure S1. According to the design conditions specified by the DoE, the products were analyzed by PLM.

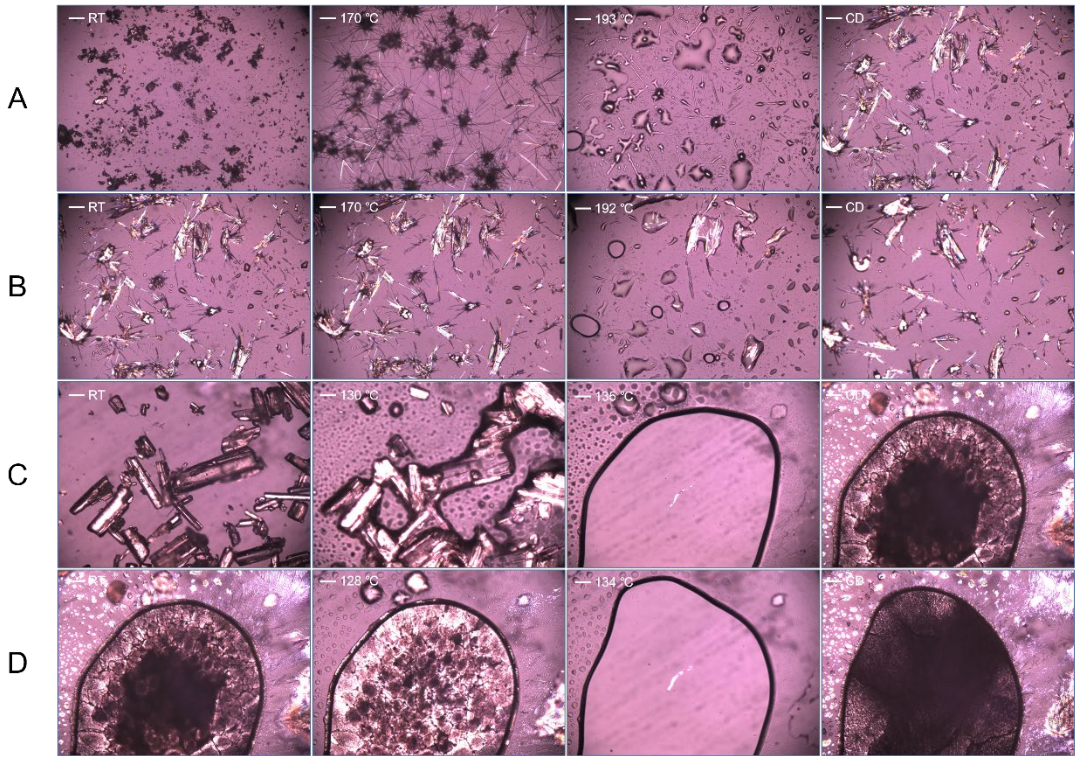

Figure S2. PLM images of pure materials. (A&B) show CBZ during a heating-cooling-reheating cycle; (C&D) show NIC during a heating-cooling-reheating cycle.
